# Editorial: Expert opinions on the relationship between microglia, glia, and Alzheimer's disease: 2022

**DOI:** 10.3389/fnagi.2023.1278808

**Published:** 2023-09-12

**Authors:** Angela Gomez-Arboledas, Helene Eliane Hirbec

**Affiliations:** ^1^Institute for Memory Impairments and Neurological Disorders (UCI MIND), University of California, Irvine, Irvine, CA, United States; ^2^Institute of Functional Genomics, CNRS, INSERM, University of Montpellier, Montpellier, France

**Keywords:** microglia, glia, Alzheimer's disease, APOE, astrocytes

Alzheimer's disease (AD) is the most prevalent form of dementia, accounting for 60–80% of cases. Although initially described more than 100 years ago, the pathogenic mechanisms underlying AD are very complex and remain poorly understood. The accumulation of amyloid plaques and neurofibrillary tangles is the key histopathological hallmark of the disease. The amyloid cascade, in which the accumulation of Aß peptides results from their overproduction or decreased clearance, has been shown to be an early event in AD and has thus been the focus of intense research. However, years of clinical failures prompted the emergence of alternative hypotheses that are not mutually exclusive.

Remarkably, genetic studies have highlighted the important role of glial cells, which are now recognized for their multifaceted contribution to maintaining neuronal health and function, in the pathogenesis of AD. As a result, an important area of investigation revolves around the intricate interplay between microglia, glia, and Alzheimer's disease, along with other related dementias. Unraveling the precise impact exerted by glial cells on the progression of AD is challenging. Among the glial cells, microglia have received significant attention due to their potential involvement in the pathogenesis of Alzheimer's disease. In fact, it has been postulated that microglia may have a dual role in the pathogenesis of the disease: they are protective in the early stages, while they drive synaptic loss and neuroinflammatory/neurodegenerative processes later on Hansen et al. (2018). The other glial cells are not at rest, and the involvement of astrocytes and oligodendrocytes in disease progression is becoming increasingly evident. Furthermore, the intimate relationship between all brain cells, including those of the vasculature, now appears to be the key to elucidating the pathological puzzle of AD.

Therefore, there is a pressing necessity for a comprehensive exploration to elucidate the relationship between glial cells and Alzheimer's disease. Our aim with this Research Topic was to provide a critical evaluation of the state of research in the field of glial cells and Alzheimer's disease.

In addition to the key role of microglial cells during the onset and progression of Alzheimer's disease, there has been recent interest in unraveling the role of astrocytes in neurodegenerative diseases. Although it is now well-established that astrocytes are essential for maintaining brain homeostasis and that astrocytic dysfunction contributes to the pathogenesis of AD, the specific mechanism remains elusive. In this context, Elsworthy et al. have nicely summarized the current literature on the potential involvement of astrocytic ADAM10 (α-disintegrin and metalloproteinase 10) in AD pathology and underscored the need for additional evidence to further comprehend the multifunctional role of these glial cells during disease progression. Continuing with the involvement of astrocytes in AD, the opinion paper by Milinkeviciute and Green not only dives into the role of clusterin (CLU) (mainly expressed by astrocytes) in Alzheimer's disease but also highlights the necessity for novel and improved models focusing on the different CLU isoforms in order to elucidate the function of this genetic risk factor for AD. Interestingly, Bonaterra-Pastra et al. performed a multicentric cohort study and showed that various CLU SNPs were associated with several Cerebral Amyloid Angiopathy (CAA) markers. They also observed an association between CLU SNPs and APOE protective genotypes with higher levels of ApoJ and ApoE in circulating lipoproteins, supporting the important role of ApoE/J in cerebral β-amyloidosis. Both CLU and APOE are important factors in the transport and metabolism of lipids (including cholesterol). In the brain, they may also modulate Aß aggregation and deposition. In this vein, Sharp et al. explored the interesting hypothesis that white matter injury, which is recognized as an important component of AD neuropathology, may contribute to cholesterol dysmetabolism, thereby increasing Aß deposition, which in turn may lead to white matter lesions, setting off a vicious injury cycle. The Blood-Brain Barrier (BBB), which is meant to protect the brain from systemic blood circulation, is another brain component that is affected in the early stages of AD. After reviewing known BBB dysfunction in AD, Sousa et al. highlighted how it may represent both a therapeutic target and a therapeutic vehicle in the treatment of the disease.

Because AD affects one of humanity's most important abilities—the ability to reason—and because the management of the disease will weigh even more heavily on our economies in the years to come, the need for effective treatments that can halt or even slow the disease is becoming ever more pressing. Exploring alternative hypotheses and integrating them with current knowledge is both a challenge and an opportunity.

## Author contributions

AG-A: Writing—original draft, Writing—review and editing. HH: Writing—original draft, Writing—review and editing.
